# P01-002 – Comparison between different colchicines responders

**DOI:** 10.1186/1546-0096-11-S1-A6

**Published:** 2013-11-08

**Authors:** ZB Özçakar, AH Elhan, F Yalcinkaya

**Affiliations:** 1 Ankara University, Pediatric Rheumatology, Turkey; 2 Biostatistics, Ankara University, Ankara, Turkey

## Introduction

Familial Mediterranean fever (FMF) is an autosomal recessive disease, characterized by recurrent, self-limited attacks of fever with serositis involving the peritoneum, pleura and joints; and colchicine is its universal treatment.

## Objectives

To explore whether the demographic and clinical features of FMF patients with different colchicine response vary or not.

## Methods

Files of patients who had been seen in our department (during routine follow-up visits) between January 2009 and January 2013 were retrospectively evaluated.

## Results

The study group comprised 221 FMF patients (116F, 105M) with a mean age of 12.7±5.3 years. Mean duration of colchicine use was 58.9±45.3 months. Patients were divided into two groups according to their colchicine response; Group I (n=131) included patients with no attacks after colchicine and Group II (n=90) patients with partial or no response to colchicine. Mean age, sex, age at disease onset, age at colchicine onset, family history of FMF, attack frequency, attack duration, clinical features during attacks, duration of colchicine use and M694V carriage were similar between the groups. Final colchicine doses, disease severity scores, acute phase reactant levels (during attack free period) were significantly higher in Group II when compared with those of Group I (p<0.5).

## Conclusion

Colchicine response seems to be related with disease severity scores and acute phase reactant levels (during attack free periods) in FMF patients.

## Disclosure of interest

None declared

